# Supplementary material for: Modeling neonatal immune response to B. pertussis identifies early B cell activation and differentiation
Source: PLoS Pathog. 2026 Apr 22;22(4):e1014163. doi: 10.1371/journal.ppat.1014163 (PMC13167031; doi:10.1371/journal.ppat.1014163)
Supplement: S1 Fig — (A) Cord blood (CB, n = 8) and adult blood (AB, n = 9) from healthy donors were stimulated ex vivo with B. pertussis (isolate FR4930, 1.75x10^7^ to 4.2x10^8^ CFU/mL). Analytes were measured in whole blood plasma/supernatant after 22 hours of infection using a 27-analytes panel (Human Luminex Assay R&D Systems, Minneapolis). In heatmaps, cytokines/ chemokines are expressed in pg/mL and log transformed, with blue to red colors representing lower to higher expression, respectively. On the x-axis, blood donors are organized by groups and by increasing concentrations of B. pertussis (CFU/mL), and on the y-axis, cytokines/chemokines are displayed following hierarchical clustering. Heatmaps were created using Qlucore OMICS explorer 3.7. The heatmap displayed all analytes secreted in non-stimulated (NS) whole blood control (CTRL) samples (both NS CB and AB, in black) and in stimulated AB (in blue) and CB (in red) samples. (B) Cell death rate in AB (blue histograms) and CB (red histograms) after stimulation (FR4930) with a range of bacterial inoculum (in CFU/mL, x-axis). Cell death rate is represented in means and standard deviation of three experiments. (DOCX) [file ppat.1014163.s001.docx]

**
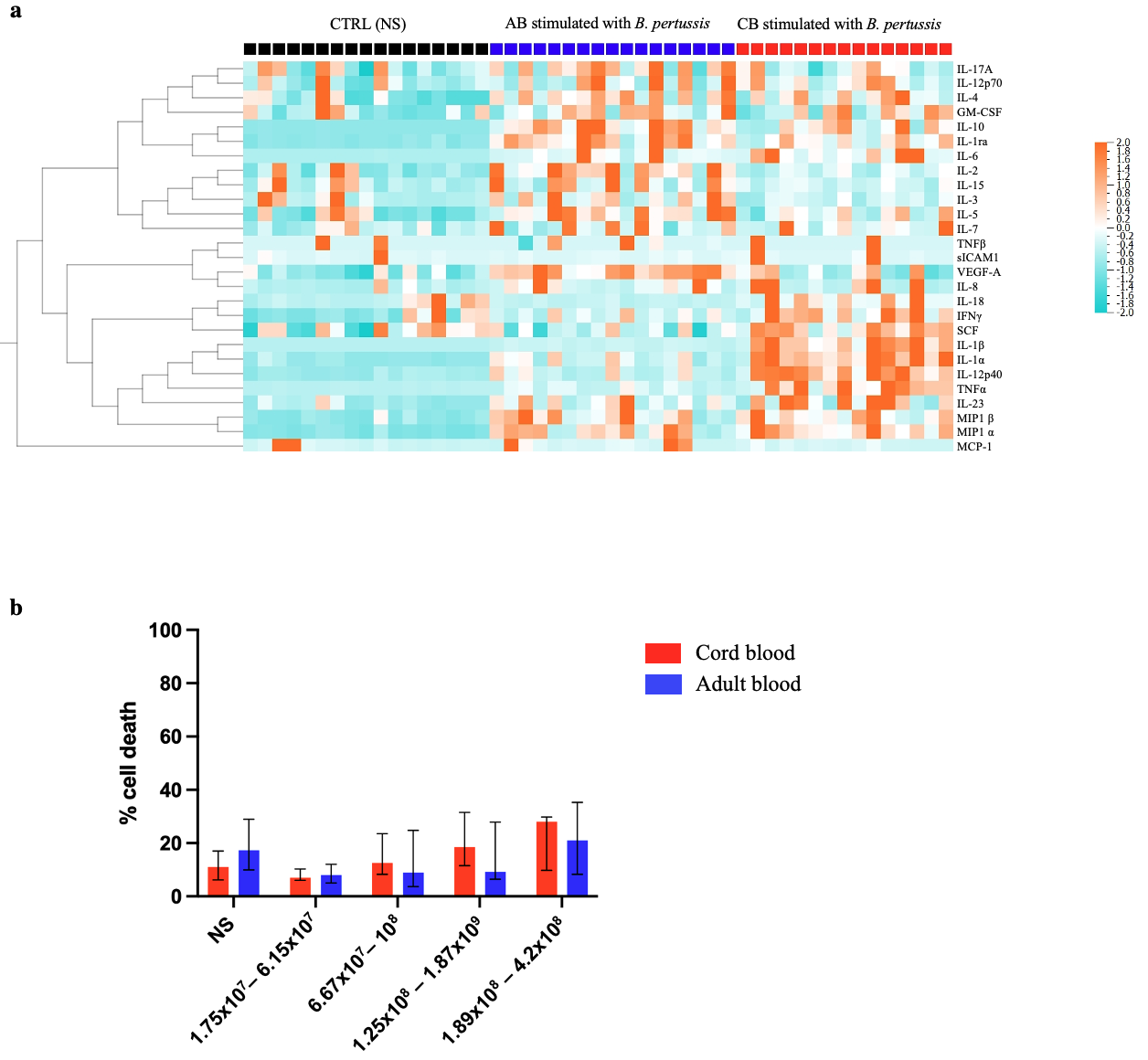
**

**B**

## **S1 Fig. Cytokine/chemokine levels in plasma samples of AB and CB after stimulation with *B. pertussis* and cell death induced by *B. pertussis*.**
