# Supplementary figures and images for: Modeling neonatal immune response to B. pertussis identifies early B cell activation and differentiation
Source: PLoS Pathog. 2026 Apr 22;22(4):e1014163. doi: 10.1371/journal.ppat.1014163 (PMC13167031; doi:10.1371/journal.ppat.1014163)

**
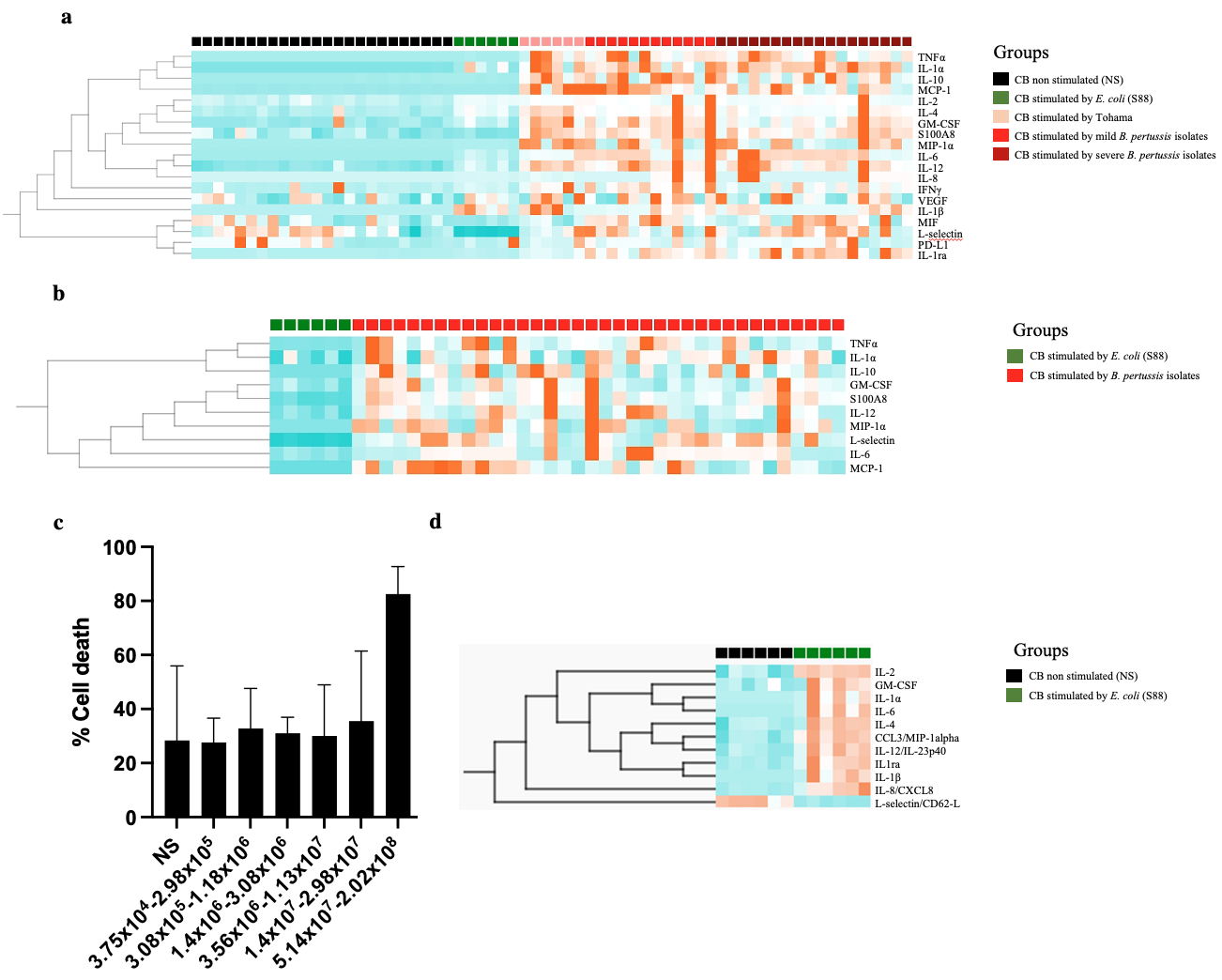
**

**A**

**D**

**C**

**B**

**S2 Fig. Analyses of cytokine/chemokine profiles of CB specific to *B. pertussis*.**

Supplement: S2 Fig — Cord blood (CB) (n = 24) from healthy donors were stimulated ex vivo with different B. pertussis isolates (Tohama, B. pertussis isolates FR5730, FR6440, FR4930, FR5333, FR5862; range 2.06x10^7^ to 3.5x10^8^ CFU/mL) and by E. coli (S88; 1.93x10^6^ to 6.725x10^7^ CFU/mL). Analytes were measured in CB plasma/supernatants after 22 hours of infection using a panel of 19 cytokines/chemokines and growth factors (Human Luminex Assay R&D Systems, Minneapolis). Cytokines are expressed as pg/mL and log transformed with blue to red colors representing lower to higher expression, respectively. (A) The heatmap represents a hierarchical clustering of all the cytokines/ chemokines and growth factors secreted in non-stimulated (NS) CB samples (in black) versus in CB samples stimulated by E. coli (in green), and by different B. pertussis isolates: Tohama (in pink), isolates from mild clinical forms (in red) and isolates from severe clinical forms (in dark red) (adjusted p-value = 1). (B) The heatmap represents a hierarchical clustering of all the cytokines/ chemokines and growth factors differentially secreted in CB samples stimulated by E. coli (in green; S88: 1.93x10^6^ to 6.725x10^7^ CFU/mL)) and by B. pertussis isolates (in red; Tohama, FR5730, FR6440, FR4930, FR5333, FR5862; 2.06x10^7^ to 3.5x10^8^ CFU/mL) with an adjusted p-value < 0.05. (C) Cell death following CB stimulation with E. coli with a range of bacterial inoculum (in CFU/mL; x-axis). Cell death rate (%) is represented in mean and standard deviation. Data representative of three experiments. (D) Cytokine release following ex vivo infection of CB samples (n = 6) with E. coli (strain S88, 1.93x10^6^ to 6.725x10^7^ CFU/mL). Analytes were measured in CB plasma/supernatants after 22 hours of infection using a 19-analyte panel (Human Luminex Assay R&D Systems, Minneapolis). Cytokines/chemokines are expressed as pg/mL and log transformed with blue to red colors representing lower to higher expression, respectively. The heatmap represents a hier [file ppat.1014163.s002.docx]

**
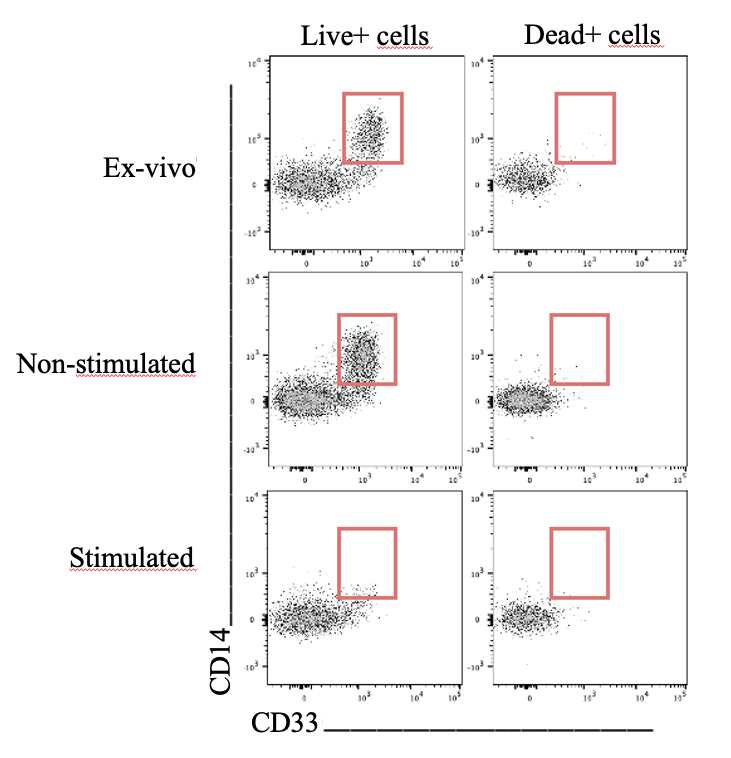
**

**S4 Fig. mMDSCs loss of detection after stimulation with *B. pertussis.***

Supplement: S4 Fig — Representative spectral-cytometry plots showing the detection of mMDSCs in CB based on their expression of CD14 and CD33 markers, across the different experimental conditions, and pre-gated either way on Live+ cells or Dead+ cells. (DOCX) [file ppat.1014163.s004.docx]

**
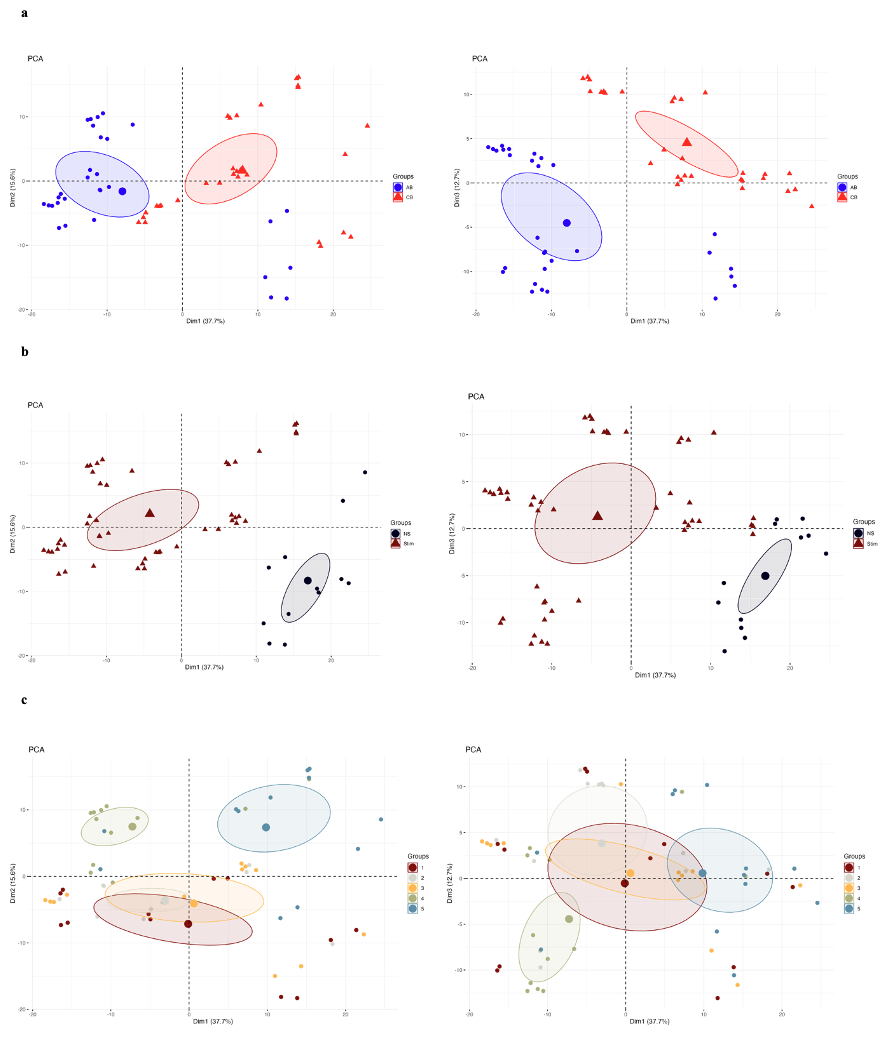
**

**C**

**B**

**A**

**S6 Fig. Principal Component Analyses (PCA) of the gene expression profiles.**

Supplement: S6 Fig — The plot shows the first three PCA axes based on all studied genes, with point colors representing gene groups. Each axis label includes the percentage of inertia, indicating the information captured by that axis. (A) Comparison of gene expression between cord blood (CB) and adult blood (AB) gene groups. (B) Comparison of gene expression between B. pertussis (FR4930), non-stimulated (NS) and stimulated gene groups.(C) Comparison across five independent experiments to assess batch effects. (DOCX) [file ppat.1014163.s006.docx]
