# Supplementary material for: Modeling neonatal immune response to B. pertussis identifies early B cell activation and differentiation
Source: PLoS Pathog. 2026 Apr 22;22(4):e1014163. doi: 10.1371/journal.ppat.1014163 (PMC13167031; doi:10.1371/journal.ppat.1014163)
Supplement: S3 Fig — (A) List of antibody’s surface-protein-targets and associated immune cell subsets used for spectral-cytometry analysis. (B) Representative flow plots and gating strategy to identify major immune cell subsets in CB. (C) Representative t-distributed stochastic neighbor embedding (t-SNE) plots from a spectral-immunophenotyping analysis of concatenated CB samples. Each dot represents an immune cell, and, for each plot, red-labeled cells correspond to the indicated immune cell compartment as determined by the gating strategy presented in (B). (DOCX) [file ppat.1014163.s003.docx]

**S3 Fig. Flow gating strategies and cell populations identification.**
