# Supplementary material for: Modeling neonatal immune response to B. pertussis identifies early B cell activation and differentiation
Source: PLoS Pathog. 2026 Apr 22;22(4):e1014163. doi: 10.1371/journal.ppat.1014163 (PMC13167031; doi:10.1371/journal.ppat.1014163)
Supplement: S5 Fig — (A) Representative spectral-cytometry plots showing the expression levels of CD25 and HLA-DR on B cells from AB (blue) or CB (red), across the different experimental conditions and at different time. (B) Representative spectral-cytometry plots showing the expression levels of CD25 and CD14 on B cells from whole-CB and PBMC from CB in non-stimulated (NS) condition or following stimulation with B. pertussis for 22 hours. (DOCX) [file ppat.1014163.s005.docx]

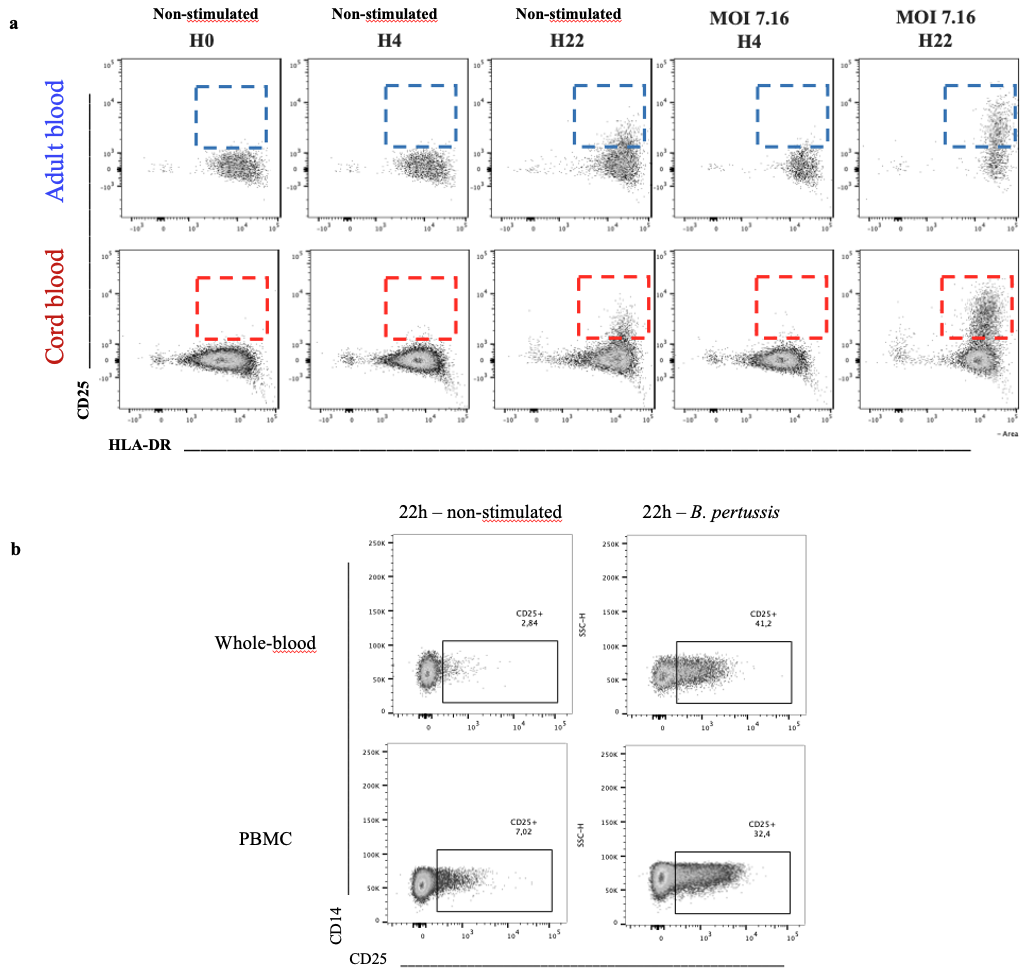


**B**

**A**

**S5 Fig. Representative flow-plots of CD25+ B cells across the different experimental conditions of infection.**
