# Supplementary material for: Modeling neonatal immune response to B. pertussis identifies early B cell activation and differentiation
Source: PLoS Pathog. 2026 Apr 22;22(4):e1014163. doi: 10.1371/journal.ppat.1014163 (PMC13167031; doi:10.1371/journal.ppat.1014163)
Supplement: S1 Table — (DOCX) [file ppat.1014163.s007.docx]

**S1 Table. Characteristics of selected *B. pertussis* isolates.**

| **Isolate number** | **Year of collection** | **FIM serotype** | **FHA production** | **PRN production** | ***ptxP* allele** | ***fim* allele** | **Associated clinical form*** |
| --- | --- | --- | --- | --- | --- | --- | --- |
| FR4930 | 2011 | FIM3 | + | + | *ptxP21* | *fim3-2* | Hospitalized in ICU |
| FR5333 | 2012 | FIM3 | + | + | *ptxP3* | *fim3-1* | Hospitalized in ICU |
| FR5730 | 2013 | FIM3 | + | + | *ptxP3* | *fim3-2* | Not hospitalized |
| FR5862 | 2014 | FIM2 | + | + | *ptxP3* | *fim3-1* | Hospitalized in ICU |
| FR6440 | 2018 | FIM2 | + | + | *ptxP3* | *fim3-1* | Not hospitalized |
| Tohama | 1954 | FIM2 | + | + | *ptxP1* | *fim3-1* | Reference strain N/A |
|  | | | |  |  |  |  |

*All strains were isolated from infants < 6 months old. FIM: Fimbriae; FHA: Filamentous Hemagglutinin; PRN: Pertactin; ICU: Intensive care unit; N/A: not applicable
