# Supplementary material for: Modeling neonatal immune response to B. pertussis identifies early B cell activation and differentiation
Source: PLoS Pathog. 2026 Apr 22;22(4):e1014163. doi: 10.1371/journal.ppat.1014163 (PMC13167031; doi:10.1371/journal.ppat.1014163)
Supplement: S2 Table — (DOCX) [file ppat.1014163.s008.docx]

**S2 Table. Eligibility criteria for cord blood and adult blood donations.**

| **Donor type** | **Criteria** |
| --- | --- |
| Cord blood | - Healthy pregnancy without major complications - Age between 18 and 40 - No infectious diseases (HIV, hepatitis B/C, syphilis) - No recent travel to high-risk areas for transmissible diseases - No history of cancer or autoimmune diseases - No drug use or high-risk behaviors |
| Adult blood | - Good health - No recent infection or vaccination - No ongoing antibiotic treatment - No recent travel to countries with infectious risk - No recent tattoage or piercings - HIV, hepatitis B or C, HTLV infections - History of certain cancers or blood diseases - Risk behaviors (e.g., intravenous drug use) - Chronic diseases affecting blood or immunity |
