# Supplementary material for: Modeling neonatal immune response to B. pertussis identifies early B cell activation and differentiation
Source: PLoS Pathog. 2026 Apr 22;22(4):e1014163. doi: 10.1371/journal.ppat.1014163 (PMC13167031; doi:10.1371/journal.ppat.1014163)
Supplement: S3 Table — (DOCX) [file ppat.1014163.s009.docx]

**S3 Table. Cell count for whole blood stimulation***

| Experiment | Blood donor | Cell count | Bacteria count |
| --- | --- | --- | --- |
| 07/12/2018 | A0 | 4.00 10^6 | 4.00 10^7 |
| 07/12/2018 | A0 | 4.00 10^6 | 2.80 10^8 |
| 14/12/2018 | A1 | 1.50 10^6 | 1.50 10^7 |
| 14/12/2018 | A1 | 1.50 10^6 | 7.50 10^7 |
| 20/12/2018 | A2 | 7.00 10^5 | 1.50 10^7 |
| 20/12/2018 | A2 | 7.00 10^5 | 7.50 10^7 |
| 28/12/2018 | A3 | 1.40 10^6 | 1.40 10^7 |
| 28/12/2018 | A3 | 1.40 10^6 | 7.0 10^7 |
| 11/01/2019 | A4 | 1.08 10^6 | 1.64 10^7 |
| 11/01/2019 | A4 | 1.08 10^6 | 8.25 10^7 |
| 17/01/2019 | A5 | 1.30 10^6 | 3.12 10^7 |
| 17/01/2019 | A5 | 1.30 10^6 | 1.56 10^8 |
| 24/01/2019 | A6 | 1.30 10^6 | 2.31 10^7 |
| 24/01/2019 | A6 | 1.30 10^6 | 1.18 10^8 |
| 31/01/2019 | A7 | 7.00 10^5 | 2.46 10^7 |
| 31/01/2019 | A7 | 7.00 10^5 | 5.17 10^7 |
| 28/02/2019 | A11 | 8.00 10^5 | 5.52 10^7 |
| 28/02/2019 | A11 | 8.00 10^5 | 2.76 10^7 |
| 14/12/2018 | C0 | 1.50 10^6 | 1.50 10^07 |
| 14/12/2018 | C0 | 1.50 10^6 | 7.50 10^7 |
| 28/12/2018 | C1 | 9.0 10^5 | 1.40 10^7 |
| 28/12/2018 | C1 | 9.0 10^5 | 7.00 10^7 |
| 11/01/2019 | C2 | 2.60 10^6 | 4.10 10^7 |
| 11/01/2019 | C2 | 2.60 10^6 | 2.05 10^8 |
| 17/01/2019 | C3 | 1.40 10^6 | 3.36 10^7 |
| 17/01/2019 | C3 | 1.40 10^6 | 1.68 10^8 |
| 24/01/2019 | C4 | 1.50 10^6 | 2.67 10^7 |
| 24/01/2019 | C4 | 1.50 10^6 | 1.34 10^8 |
| 07/02/2019 | C6 | 1.70 10^6 | 5.57 10^7 |
| 07/02/2019 | C6 | 1.70 10^6 | 1.38 10^8 |
| 28/02/2019 | C10 | 1.10 10^6 | 7.59 10^7 |
| 28/02/2019 | C10 | 1.10 10^6 | 3.80 10^7 |
| 28/02/2019 | C11 | 1.70 10^6 | 1.15 10^8 |
| 28/02/2019 | C11 | 1.70 10^6 | 5.87 10^7 |

A: adult blood and C: cord blood (number corresponding to each individual donor)

*Median concentration of cells from adult (n=9) or cord blood (n=8) samples that do not differ significantly (Mann–Whitney, p = 0.12), and the overall variance indicates a similar dispersion across samples
