# Supplementary material for: Modeling neonatal immune response to B. pertussis identifies early B cell activation and differentiation
Source: PLoS Pathog. 2026 Apr 22;22(4):e1014163. doi: 10.1371/journal.ppat.1014163 (PMC13167031; doi:10.1371/journal.ppat.1014163)
Supplement: S4 Table — (DOCX) [file ppat.1014163.s010.docx]

**S4 Table. Luminex Panels.**

**Table A. 27-analyte panel.**

| **Function** | **Analyte** |
| --- | --- |
| Pro-inflammatory analytes | IL-1α |
|  | IL-1β |
|  | IL-12/23 p40 |
|  | IL-15 |
|  | IL-18 |
|  | IL-2 |
|  | IL-3 |
|  | IL-4 |
|  | IL-5 |
|  | IL-6 |
|  | IL-7 |
|  | IL-12p70 |
|  | IL-17A |
|  | IL-23 |
|  | IFNγ |
|  | TNFα |
|  | TNFβ |
| Anti-inflammatory analytes | IL-1ra |
|  | IL-10 |
| Chemokines | IL-8 (CXCL8) |
|  | MCP-1 (CCL2) |
|  | MIP-1α (CCL3) |
|  | MIP-1β (CCL4) |
| Adhesins | ICAM-1 |
| Growth factors | SCF |
|  | GM-CSF |
|  | VEGF-A |

**Table B. 19 analyte panel.**

| **Function** | **Analyte** |
| --- | --- |
| Pro-inflammatory analytes | IL-1α |
|  | IL-1β |
|  | IL-12p40 |
|  | IL-2 |
|  | IL-4 |
|  | IL-6 |
|  | TNFα |
|  | IFNγ |
|  | S100A8 |
| Anti-inflammatory analytes | IL-1ra |
|  | IL-10 |
|  | PD-L1 |
| Chemokines | MIP-1α |
|  | MCP-1 |
|  | IL-8 |
|  | MIF |
| Adhesins | L-Selectin |
| Growth factors | VEGF-A |
|  | GM-CSF |
