# Supplementary material for: Modeling neonatal immune response to B. pertussis identifies early B cell activation and differentiation
Source: PLoS Pathog. 2026 Apr 22;22(4):e1014163. doi: 10.1371/journal.ppat.1014163 (PMC13167031; doi:10.1371/journal.ppat.1014163)
Supplement: S5 Table — (DOCX) [file ppat.1014163.s011.docx]

**S5 Table. Antibodies and dyes used for flow cytometry.**

**S5A. Antibodies and dyes used to investigate immune cell populations following
*B. pertussis ex vivo* stimulation.**

| **Product** | **RRID citation*** |
| --- | --- |
| PerCP Anti-Human CD8a | BioLegend Cat# 300921, RRID:AB_1575076 |
| PE/Cy5 Anti-Human CD3 | BioLegend Cat# 300309, RRID:AB_314045 |
| Brilliant Violet 650 Anti-Human CD4 | BioLegend Cat# 300535, RRID:AB_2561351 |
| Brilliant Violet 711 Anti-Human CD56 | BioLegend Cat# 362541, RRID:AB_2565919 |
| PE/Cy7 Anti-Human CD11c | BioLegend Cat# 301607, RRID:AB_389350 |
| Brilliant Violet 605^TM^ Anti-Human CD123 | BioLegend Cat# 306025, RRID:AB_2562115 |
| Brilliant Violet 570^TM^ Anti-Human CD127 | BioLegend Cat# 351307, RRID:AB_10900064 |
| CD19 BV 750 | BioLegend Cat# 302261, RRID:AB_2734257 |
| FITC Anti-Human CD66b | BioLegend Cat# 305103, RRID:AB_314495 |
| PE Anti-Human CD25 | BioLegend Cat# 302605, RRID:AB_314275 |
| PE/Dazzle 594 Anti-Human HLA-DR | BioLegend Cat# 307653, RRID:AB_2563645 |
| Brilliant Violet 510 Anti-Human CD33 | BioLegend Cat# 303427, RRID:AB_2650887 |
| BV480 Mouse Anti-Human CD45 | BD Biosciences Cat# 566115, RRID:AB_2739517 |
| Brilliant Violet 650 Anti-Human CD71 | BioLegend Cat# 334115, RRID:AB_2687102 |
| PE/Cy5 Anti-Human CD235 | BioLegend Cat# 306605, RRID:AB_314623 |
| Anti-Human CD14 Alexa Fluor 523 | Thermo Fisher Scientific Cat# 58-0149-41, RRID:AB_11218093 |
| Live/Dead^TM^ Fixable Violet Dead Cell Stain Kit (405 nm excitation) | Thermo Fisher Scientific Cat# L34964, RRID: N/A |

* RRID = Research resource identifier

**S5B. Antibodies used to investigate CD25+ B cells.**

| **Product** | **RRID citation** |
| --- | --- |
| PE/cyanine5 Anti-Human CD56 | (BioLegend Cat# 362515, RRID:AB_2564088) |
| PE/cyanine5 Anti-Human CD16 | (BioLegend Cat# 302009, RRID:AB_314209) |
| PE/cyanine5 Anti-Human CD14 | (BioLegend Cat# 301863, RRID:AB_2860766) |
| Brilliant Violet 711 Anti-Human CD24 | (BioLegend Cat# 311135, RRID:AB_2566578) |
| PE Anti-Human CD25 | (BioLegend Cat# 302606, RRID:AB_314276) |
| PR/cyanine7 Anti-Human IgG | (BioLegend Cat# 410721, RRID:AB_2750226) |
| Brilliant Violet 421 Anti-Human IgM | (BioLegend Cat# 314515, RRID:AB_10895748) |
| FITC Anti-Human CD5 | (BioLegend Cat# 364021, RRID:AB_2566247) |
| Brilliant Violet 510 Anti-Human CD10 | (BioLegend Cat# 312219, RRID:AB_2561722) |
| Brilliant Violet 650 Anti-Human CD71 | (BioLegend Cat# 334116, RRID:AB_2687103) |
| Zombie NIR Fixable Viability Kit | (BioLegend Cat# 423106, RRID:N/A) |
| BB700 Mouse Anti-Human IgD | (BD Biosciences Cat# 566538, RRID:AB_2744486) |
| BUV737 Mouse Anti-Human CD3 | (BD Biosciences Cat# 612753, RRID:AB_2870084) |
| BUV395 Mouse Anti-Human CD38 | (BD Biosciences Cat# 563811, RRID:AB_2744372) |
| BUV496 Mouse Anti-Human CD21 | (BD Biosciences Cat# 750614, RRID:AB_2874746) |
| Qdot 605 Anti-Human CD4 | (Thermo Fisher Scientific Cat# Q10008, RRID:AB_11180611) |
| BV-786 Anti-Human CD80 | Provided by Inserm U976 Laboratory |
| BV-510 Anti-Human CD40 | Provided by Inserm U976 Laboratory |
| FITC Anti-Human CD19 | Provided by Inserm U976 Laboratory |
| PE/Cy7 Anti-Human CD27 | Provided by Inserm U976 Laboratory |
