# Supplementary material for: Modeling neonatal immune response to B. pertussis identifies early B cell activation and differentiation
Source: PLoS Pathog. 2026 Apr 22;22(4):e1014163. doi: 10.1371/journal.ppat.1014163 (PMC13167031; doi:10.1371/journal.ppat.1014163)
Supplement: S6 Table — (DOCX) [file ppat.1014163.s012.docx]

**S6 Table. Expansion of the Nanostring Host Response panel with additional genes.**

| **Gene** | **Target** | **Full Name** |
| --- | --- | --- |
| ARG1 | TCAATGACTGAAGTGGACAGACTAGGAATTGGCAAGGTGATGGAAGAAACACTCAGCTATCTACTAGGAAGAAAGAAAAGGCCAATTCATCTAAGTTTTG | Arginase 1 |
| BTK | AGCAAAGCTGGCAAATATACAGTGTCTGTGTTTGCTAAATCCACAGGGGACCCTCAAGGGGTGATACGTCATTATGTTGTGTGTTCCACACCTCAGAGCC | Bruton tyrosine kinase |
| CD58 | GTGCTTGAGTCTCTTCCATCTCCCACACTAACTTGTGCATTGACTAATGGAAGCATTGAAGTCCAATGCATGATACCAGAGCATTACAACAGCCATCGAG | CD58 molecule |
| CD83 | ATTTTCTGTGGGCAGGCCTCGAAAACCATCACATGACCACATAGCATGAGGCCACTGCTGCTTCTCCATGGCCACCTTTTCAGCGATGTATGCAGCTATC | CD83 molecule |
| CEACAM1 | AGAGGGAGGGGTTATAGCTTCAGGAGGGAACCAGCTTCTGATAAACACAATCTGCTAGGAACTTGGGAAAGGAATCAGAGAGCTGCCCTTCAGCGATTAT | CEA cell adhesion molecule 1 |
| CEACAM8 | CGTGGCTACAACTGGTACAAAGGGGAAACAGTGGATGCCAACCGTCGAATTATAGGATATGTAATATCAAATCAACAGATTACCCCAGGGCCTGCATACA | CEA cell adhesion molecule 8 |
| CFH | GGAAAAATTGTCAGTAGTGCAATGGAACCAGATCGGGAATACCATTTTGGACAAGCAGTACGGTTTGTATGTAACTCAGGCTACAAGATTGAAGGAGATG | Complement factor H |
| CR2 | AGCCCAGTTTCACTGCCATATACTCTTCAAGGACTTTCTGAAGCCTCACTTATGAGATGCCTGAAGCCAGGCCATGGCTATAAACAATTACATGGCTCTA | Complement C3d receptor 2 |
| ICAM1 | AAATACTGAAACTTGCTGCCTATTGGGTATGCTGAGGCCCCACAGACTTACAGAAGAAGTGGCCCTCCATAGACATGTGTAGCATCAAAACACAAAGGCC | Intercellular adhesion molecule 1 |
| KITLG | AGGACTCTATTTTAAGGACTGCGGGACTTGGGTCTCATTTAGAACTTGCAGCTGATGTTGGAAGAGAAAGCACGTGTCTCAGACTGCATGTACCATTTGC | KIT ligand |
| LTA | CCCATTCTGCCTCCATTCTGACCATTTCAGGGGTCGTCACCACCTCTCCTTTGGCCATTCCAACAGCTCAAGTCTTCCCTGATCAAGTCACCGGAGCTTT | Lymphotoxin alpha |
| MMP8 | CTCTCTCCTAGAGTCCAAACCCAAATGGGCCAGTTGGATCTGATGTTCGTCAGTTCTTTACTTCTATTTCCTGGGGTACTCAGGAGGGCACACACTATAG | Matrix metallopeptidase 8 |
| NFKBIA | ATTTATTGTGCTTCGAGTGACTGACCCCAGTGGTATCCTGTGACATGTAACAGCCAGGAGTGTTAAGCGTTCAGTGATGTGGGGTGAAAAGTTACTACCT | NFKB inhibitor alpha |
| S100A8 | GTTAACTTCCAGGAGTTCCTCATTCTGGTGATAAAGATGGGCGTGGCAGCCCACAAAAAAAGCCATGAAGAAAGCCACAAAGAGTAGCTGAGTTACTGGG | S100 calcium binding protein A8 |
| S100A9 | TGAACCAGGGGGAATTCAAAGAGCTGGTGCGAAAAGATCTGCAAAATTTTCTCAAGAAGGAGAATAAGAATGAAAAGGTCATAGAACACATCATGGAGGA | S100 calcium binding protein A9 |
| TIRAP | ACCCACACATGCGAGTGACAGTGGCAGTAGTCGCTGGAGCAAAGACTATGACGTCTGCGTGTGCCACAGTGAGGAAGACCTGGTGGCCGCCCAGGACCTG | TIR domain containing adaptor protein |
| TNFAIP2 | CAAAGGCCACCTGAGCGCTATCCTGGCCATCAAGGGGAACCTATCCAACAGTGAGGTCAAGCGCATCCGGAGCATCTTGGACGTCAGCATGGGGGCGCAG | TNF alpha induced protein 2 |
| TNFAIP3 | TCAGGGAAAATGGACGTATTCAGAGAGTGTTTGTAGTTCATGGTTTTTCCCTACCTGCCCGGTTCCTTTCCTGAGGACCCGGCAGAAATGCAGAACCATC | TNF alpha induced protein 3 |
| TREM1 | GTTTACTGCGCGTCCGAATGGTCAACCTTCAAGTGGAAGATTCTGGACTGTATCAGTGTGTGATCTACCAGCCTCCCAAGGAGCCTCACATGCTGTTCGA | Triggering receptor expressed on myeloid cells 1 |
| TREM2 | GGCCACCAGGACTCCTTGTTCTGCTCTGGCAAGAGACTACTCTGCCTGAACACTGCTTCTCCTGGACCCTGGAAGCAGGGACTGGTTGAGGGAGTGGGGA | Triggering receptor expressed on myeloid cells 2 |
