# Supplementary material for: Modeling neonatal immune response to B. pertussis identifies early B cell activation and differentiation
Source: PLoS Pathog. 2026 Apr 22;22(4):e1014163. doi: 10.1371/journal.ppat.1014163 (PMC13167031; doi:10.1371/journal.ppat.1014163)
Supplement: Attachment — Submitted filename: Response to Reviewers_PPATHOGENS-D-25-01905_Final_0190126.docx [file ppat.1014163.s015.docx]

**PPATHOGENS-D-25-01905**

**Reviewers' comments for the article “Modeling neonatal immune response to B. pertussis identifies early B cell activation and differentiation »**

We thank the Reviewers and the Editorial Board for their careful review of our manuscript and their constructive criticisms. We addressed each concern raised and revised our manuscript accordingly.

**Part I – Summary**

**Reviewer #1**

Bordetella pertussis infections are resurging despite global vaccination efforts. As the authors highlight, Bp infection is more severe in infants who have yet to receive any vaccination. In an effort to explore these differences, the authors develop an ex vivo infection model taking both adult blood and cord blood and treating them with different strains of Bp, highlighting differences between the immune responses in the different sources of blood. The authors highlight that while the cord blood is naturally suppressive in nature, that after Bp infection in their ex vivo model, there is a hyperinflammatory signature in cord blood. Similar in both blood sources was the activation of CD25+ B cell responses. Although this work could make a significant contribution to the field, there are several inconsistencies, unclear points, and **aspects where the data lack sufficient context or relevance.**

**Reviewer #2:** The manuscript “Modeling neonatal immune response to B. pertussis identifies early B cell activation and differentiation” by Matczak et al. utilizes an ex vivo whole-blood infection model of cord blood and adult blood to provide valuable new knowledge on age-specific immune responses to B. pertussis. This study is novel and generated information that will be of great interest in the field. The applied studies are robust, and the manuscript is clearly written. More information on experimental conditions is needed, and additional improvements are suggested.

Response Part I: We thank the reviewers for their general comments, and hope that our detailed responses and the additional information we have provided address the reviewers’ concerns and clarify the data and context of our study.

**Part II – Major Issues: Key Experiments Required for Acceptance**

**Reviewer #1:**

Comment 1: It is noted that the authors measured the IgG responses to PT to determine whether the patient had recent exposure or an active infection. **Vaccination history of the maternal parent** should be included, as well as immunization history. Even though low titers describe an individual that is not immunized, literature shows that prior immunization can dictate the immune responses, specifically the T cell responses. If this information can not be obtained from the patients, **this should be highlighted** in the discussion, as this could potentially impact the results. Additionally, the impact of the study can be significantly improved if cohorts who are both aPV and wPV immunized could be examined or if maternal immunization strategies had any impact on the cord blood.

Response: We thank the Reviewer for this thoughtful comment. Unfortunately, the vaccination history of the donors was not available in our dataset, as the cord blood as well as adult blood samples were obtained from anonymous donors. The only details available derived from eligibility criteria for donation, which included good health, no drug use, no recent travel with infectious risk and no ongoing antibiotic treatment. We added these details in the Supporting information files **(S2 Table)**. Negative serology only suggests the absence of recent exposure or immunization (natural or vaccine-induced), and we agree that prior immunization may influence immune responses, including T cell profiles. We have now acknowledged this limitation in the Discussion section: **“Another limitation of our study is the lack of information on vaccination history of the donors, as both CB and AB samples were obtained from anonymous individuals. Although the negative serology suggests an absence of recent natural exposure or vaccine-induced immunization, previous vaccinations could still have influenced immune responses, particularly B and T cell–mediated ones. Future studies including detailed immunization data or comparing cohorts with different vaccine backgrounds would help clarify these effects on maternal and cord blood immunity. »**

Comment 2: **Further details of the strains are needed here**. The information that is provided essentially highlights that they are the same. Additionally, countries that aPV are experiencing many PRN deficient strains.

Response: The characteristics of the strains used in this study are detailed in **S1 Table**. All strains express PRN, as the objective of the study was not to compare immune responses according to PRN expression, which may indeed have an impact (PMID: 34132167). Except for the reference strain Tohama, all isolates are recent circulating strains obtained from patients (for whom disease severity was documented in cytokineS1 Table) and show few phenotypic differences, particularly regarding FIM expression.

To address the reviewer request, in the revised manuscript, we also included genotypic data (*PtxP* and *fim* alleles).

Comment 3: More information is needed from the individuals that the blood was collected from. For example, any medication or drugs that the individual is on needs to be noted.

Response: As mentioned above, data from the donors were not available as the cord blood as well as adult blood samples were obtained anonymously. The only details available derived from eligibility criteria for donation. We added these details in the Supporting information files (**S2 Table).**

Comment 4: From the methods that are described, **a volume of blood was used, not a cell count**. I think it is necessary to have some sort of normalization of the cell number and types when presenting this data. This also makes the data reproducible when highlighting the amount of bacteria used. Furthermore, the manuscript as a whole would have a greater impact if the cell type responsible for the cytokine profiles that are observed were determined. What cells are specifically contributing to the cytokine profiles. Intracellular flow cytometry on the specific innate and adaptive immune cells would determine the relative contribution of each cell type.

Response: We thank the Reviewer for this valuable comment. Indeed, cell numbers were measured before each experiment and were used for normalization. As the experiments were performed in whole blood, we did not normalize cell numbers prior to stimulation. Instead, the bacterial inoculum (CFU) was adjusted to cell numbers, and we subsequently verified that there was no significant variation in relevant cell numbers across experiments. We have clarified the Methods section: **“After sampling few milliliters of each blood sample and red blood cell lysis, cells were counted following Trypan Blue Stain coloration (Biorad TC20). From an estimated concentration of 1.0 x10^9^ CFU/ml of *B. pertussis* corresponding to a suspension of OD_650_=1 (laboratory data), we used the volume of bacteria necessary to reach an estimated MOI from 10 to 50 (Matczak et al, Microbes and Infection, PMID: 37245862). This corresponded to a measured CFU of 1.75 x10^7^ to 4.2 x10^8^/ml”**. We also have made available in the Supporting Information a table detailing, for each experiment, the number of cells and the CFU/mL used **(S3 Table**). The number of cells per equal volume of blood did not vary substantially across samples, with a median concentration of cells from adult (n=9) or cord blood (n=8) samples that do not differ significantly (Mann–Whitney, p = 0.12), and the overall variance indicates a similar dispersion across samples.

Second, in this study, cytokine measurements were performed on secreted cytokines from a whole blood stimulation assay, which captures the integrated response of all circulating immune cells within their physiological environment. Thus, the data reflects the global cytokine response of whole blood, rather than cytokine production by specific cell subsets. Previous studies have shown that quantifying secreted cytokines in whole blood assays gives robust, reproducible, and biologically meaningful results, particularly for standardizing immune monitoring across large human cohorts (Duffy et al., Clinical Immunol. 2017, PMID: 28943400; Duffy et al., Immunity. 2014, PMID: 24656047). Compared with intracellular staining, which is ideal for identifying cytokine producing subsets, secreted cytokine analysis allows broader functional assessment of cytokine magnitude and kinetics across the circulating immune compartment while minimizing technical variability introduced during PBMC isolation as well as the use of protein trafficking inhibitors (e.g., Brefelidin A) required for intra-cellular staining.

We recognize the value of identifying the cellular sources of cytokines and agree that intracellular flow cytometry could provide additional insight. However, based on our results we chose to focus specifically on B cells, which were markedly increased following stimulation, and we further investigated these cells through transcriptomic analyses which provided much richer data sets than assessing cytokines by intracellular staining. Extending this approach to all immune cell types would have been beyond the scope of the present study. This has been noted in the *Discussion* section: “**Another limitation of our study is that cytokine measurements were based on released cytokines from whole blood stimulations, reflecting the global secretome following stimulation with *B. pertussis* rather than the contribution of individual cell subsets. We further explored B cells, whose proportion markedly increased following stimulation, through transcriptomic analyses; however, extending this to all immune cell types was beyond the scope of the present study. Future work using approaches such as intracellular flow cytometry or single-cell analyses would provide valuable complementary information on the specific cellular sources of cytokines. »**

Comment 5: Lines 72-73. Although Fig. 1 lists multiple *B. pertussis* strains, in the cytokine/chemokine analysis the authors focus on FR4930 without explaining why this strain was selected for the initial analyses. The authors should clarify.

Response: FR4930 was used for analyses as it is a clinical strain from a recent pediatric severe case, with a phenotype corresponding to currently circulating strains: it produces FHA and PRN2, like most PRN-positive isolates. We preferred to use clinical strains rather than a reference strain such as the Tohama, which we used only for control. As mentioned above, all data regarding strains are available in the Supporting Information files (**S1 Table**).

Comment 6: Fig. 2 (cytokine/chemokine analysis). The control (non-stimulated) samples display high baseline levels of several cytokines/chemokines (e.g., sICAM ~10⁴ pg/ml, IL-12p70, IL-12, IFN-γ). Moreover, IFN-γ levels appear indistinguishable between stimulated and non-stimulated conditions, yet the authors describe it as “specific to stimulation” in line 78.The authors should explain why the non-stimulated samples show such high baseline levels and discuss the biological relevance of these findings.

There also appears to be a discrepancy between the differences observed in non-stimulated versus stimulated samples in Fig 2A and the levels shown in Fig 2B. Basal cytokine/chemokine levels are not comparable between CB and AB samples in Fig 2A, but the authors do not indicate which basal levels were used to generate Fig 2B.

Response: Thank you for this remark. Indeed, the elevated concentrations of soluble ICAM, in whole blood from healthy adult donors (AB) are consistent with the literature. (Biancotto et al, Plos one, PMID: 24348989; Duffy et al., Immunity. 2014, PMID: 24656047). IL-12p70 and IFN**γ** are slightly elevated at baseline, though similar to the literature data (Biancotto et al, Plos one, PMID: 24348989) and this likely reflects the baseline levels in the plasma which are captured in the unstimulated whole blood condition. We agree that in cord blood of some donors, baseline levels of IFNγ are already elevated, and this inter-donor variability limits the differences observed between non-stimulated and stimulated conditions. We corrected in the results section, as follows: **“At baseline, ICAM levels were elevated, as previously described (Biancotto et al, Plos one, PMID: 24348989; Duffy et al., Immunity. 2014, PMID: 24656047). When cytokine concentrations from stimulated CB and AB were directly compared, 10 showed differential secretion (Fig 2B); of these, five were significantly upregulated upon stimulation: VEGF-A was found more elevated in AB whereas levels of IL-1α, IL-1β, IL-12p40 and TNF-α were higher in CB after stimulation with *B. pertussis*. IFNγ levels were higher in CB than in AB, but the stimulation-specific effect did not reach statistical significance, likely due to elevated baseline IFNγ levels and inter-donor variability.”**

The discrepancy between the differences observed in non-stimulated versus stimulated in the boxplots (former Fig 2A) and the heatmap (former Fig 2B) is due to the fact that the heatmap is computed from transformed data (cytokine-wise scaling is applied prior to visualization). This normalization step modifies the distribution of the raw measurements. For clarification, we removed Fig 2B and revised Fig 2A to more accurately reflect the complete set of data (both non-stimulated and stimulated conditions) and to better illustrate the effect of stimulation in the two blood types.

Comment 7: Fig 2, line 109. The term “expression” is not appropriate in this context. Cytokine/chemokine measurements represent secreted levels, not gene or protein expression. The wording should be corrected accordingly.

Response: We thank the Reviewer for this remark. We corrected accordingly in Results section in Fig 2 legend: **“In heatmap (B), cytokines/chemokines are expressed in pg/mL and log transformed, with blue to red colors representing lower to higher secretion respectively”**

Comment 8: Fig 2, line 110. The authors mention that samples were organized by increasing bacterial concentrations and by groups along the y-axis, but the grouping strategy and actual concentrations are not clearly indicated in the figure or in the Results section. This omission makes interpretation difficult. The authors should clarify how groups were defined and ensure that concentrations are explicitly shown in the figure. In addition, it is not clear which concentration was used to generate Fig 2A.

Response: As mentioned in response to comment 6, we removed Fig 2B, which was redundant with Fig 2A. For all Fig 2 experiments, we used concentrations of *B. pertussis* (isolate FR4930) from 1.75x10^7^ to 4.2x10^8^ CFU/mL”, as describe in the Figure Legend. For the Heatmap (Fig 2B), we clarified the figure description, as follows: “**On the x-axis, experiments are organized by blood type (blue, AB; red, CB) and by concentration levels of *B. pertussis* (left to right, from 1.75x10^7^ to 4.2x10^8^/ml CFU/mL). On the y-axis, cytokines/chemokines are displayed following hierarchical clustering**.”

Comment 9: Supplementary Fig S1. It is not indicated how cell death was evaluated, either in the figure or in the Materials and Methods. This information is essential for interpreting the results. Cytokine/chemokine-producing immune cells circulating in blood, such as monocytes and lymphocytes, can survive for more than 24 h ex vivo, whereas other populations such as neutrophils could have a much shorter lifespan (~6–17 h). Despite this, no reduction in viability is reported after 22 h post-infection. The authors should clarify how cell death was assessed and explain these observations.

Response: We thank the Reviewer for catching this up. We have now clarified the methodology used to evaluate cell death in the Materials and Methods section, as follows: “**Cell death was assessed either by Trypan Blue stain coloration using a TC20 automated cell counter (Bio-Rad) or by flow cytometry after staining with a fixable viability dye (Zombie NIR™ Fixable Viability Kit, BioLegend) following red blood cell lysis. Cell mortality was expressed as the percentage of non-viable cells (Trypan Blue–positive or Zombie NIR–positive) and reported as mean ± standard deviation from three independent experiments”.**

Regarding the viability profile, all conditions, including the non-stimulated (NS) controls, were incubated for 22 h before staining. Thus, leukocytes with very short *ex vivo* lifespans may already have progressed to late apoptosis or fragmentation and been excluded as debris during flow cytometry gating. As a result, the assay may reflect the viability of the intact leukocyte population remaining at 22 h, rather than the cumulative loss of all cells over time. Under these conditions, overall cell death remained below 30% across all *B. pertussis* concentrations, indicating that stimulation did not induce substantial additional cytotoxicity.

Comment 10: Line 90. The authors should clarify the rationale for including an E. coli strain in the Results section, as this would facilitate interpretation of the comparative findings.
Line 96. The authors state that B. pertussis induced a stronger inflammatory response compared to E. coli; however, this difference may be confounded by the higher levels of cell death observed with E. coli.

Response: For clarification we added a rationale in Results section: “**To analyze whether this cytokine response is specific to *B. pertussis* infection and whether it is strain-dependent, we investigated effect of *E. coli* stimulation (*E. coli* S88 strain) on human cord blood. The *E. coli* strain was used as a reference, as it is a Gram-negative bacterium often responsible for severe infections in young infants**”.

Regarding cell death rates, we compared those associated with *B. pertussis* and *E. coli*, and at the tested CFU levels, the rates were comparable, as mentioned in the manuscript. We selected bacterial concentrations corresponding to equivalent levels of cell death. These concentrations are mentioned in the Methods section, which was clarified: “**A range of** ***E. coli* concentration was used for CB stimulation selecting those that resulted in cell mortality levels comparable to those induced by *B. pertussis* (i.e., <30% cell mortality; concentrations from 1.93x10^6^ to 6.72x10^7^ CFU/mL; S2C Fig) and still triggered an inflammatory response (S2D Fig).**”

**Part III – Minor Issues: Editorial and Data Presentation Modifications**

**Reviewer #2:**

Comment 1: The methods do not clearly specify the volumes of blood used per well in each experiment, nor whether cell concentrations (e.g., cells per mL) were normalized across samples, citing only that 200 to 500 ul of whole blood samples were used. This is particularly important given that the study compares whole cord blood (CB) and adult blood (AB), which can differ in cell numbers. Indeed, the finding that cytokine responses in CB were more elevated at a protein level than what was observed for transcriptional responses may be due to increased cell numbers in the CB resulting in higher amounts of secreted proteins, while transcript measurements are normalized to the expression of housekeeping genes. The authors should provide additional information on the volumes of blood used per experiment and whether cell concentrations were normalized. If these factors were not controlled, a discussion of the potential impacts of differences in cell numbers per well should be added.

Response: We thank the Reviewer for this valuable comment. Regarding the volume of blood used, it was 200 μL for cytokine analyses and 500 μL for transcriptomic analyses. We added this essential detail in the Methods section. Besides, as mentioned in the response to Reviewer#1 (Comment 4), leucocyte concentrations were measured before each experiment. We have made available in the Supporting Information a table detailing, for each experiment, the number of cells and the CFU/mL used **(S3 Table**). The number of cells per equal volume of blood did not vary substantially across samples.

Comment 2: The assay used to measure cell death is not described in the methods, please add this information.

Response: We thank the Reviewer for highlighting this. As mentioned in the response of Reviewer#1 comment 9, we have now clarified this point in the Methods section.

“**Cell death was assessed either by Trypan Blue stain coloration using a TC20 automated cell counter (Bio-Rad) or by flow cytometry after staining with a fixable viability dye (Zombie NIR™ Fixable Viability Kit, BioLegend) following red blood cell lysis. Cell mortality was expressed as the percentage of non-viable cells (Trypan Blue–positive or Zombie NIR–positive) and reported as mean ± standard deviation from three independent experiments”.**

Comment 3 A discussion of the potential impact of comparing different infectious doses for *B. pertussis* and *E. coli* would improve reader comprehension.

Response: We thank the Reviewer for this comment. We added in the Discussion methods: “**This may be explained by an LPS specific hyperinflammatory response; however, it has not been observed following stimulation with *E. coli*,** **which may indicate a more specific response to *B. pertussis.* As lower CFU levels of *E. coli* were required to achieve comparable levels of cell death, which may introduce bias, further studies using *B. pertussis* LPS for stimulation will be needed to confirm our results**.”

Comment 4: The described figure annotations for Supplementary Fig S2 are incorrect (line 95-97). S2C displays cell death measures, not S2B; S2D displays E. coli-induced inflammation not S2C; S2B compares B. pertussis with E. coli, not S2D.

Response: We revised the figure captions and updated the figure numbering accordingly.

Comment 5: Information on the source (CB or AB or both) of control (NS) blood displayed in Fig 2B needs to be added.

Response: We agree with the Reviewer. In response to Comment 6 from Reviewer#1, we removed Fig 2B, which was redundant with Fig 2A. The latter Figure was improved for clarification.

Comment 6: INFg should be changed to IFNg on line 116, 287 and Fig 1.

Response: We changed subsequently.

Comment 7: Both **moMDSC and mMDSC** are used e.g. line 120 and line 125. This should be standardized.

Response: We standardized the denomination with “mMDSC”.

Comment 8: The numbers of samples per group should be given for Fig 4B, as there appear to be overlapping samples or just 1 sample was evaluated for % CD25+ B cells in AB stimulated with Tohama. If this group is n=1, statistical analysis should not be applied.

Response: We thank the Reviewer for this comment. In the Fig 4B legend, we included the exact number of samples per group. Besides, Statistical analyses were not performed on individual strains. Instead, all *B. pertussis*-stimulated samples were pooled into a single “*B. pertussis*-stimulated” group, independently of the isolate, for the purpose of comparison between the two groups “no stimulation” and “*B. pertussis*-stimulated” (AB, n=18; CB, n=23). Thus, no statistical test was conducted on the Tohama AB group alone. We have clarified this point in the Figure Legend and ensured that the pooled-group statistical analysis is clearly stated in the figure caption: **“(B) Dot plots showing the frequency of CD25⁺ B cells in adult blood (AB, blue) and cord blood (CB, red) across experimental conditions. Four *B. pertussis* isolates (FR4930, FR5730, FR5333, and Tohama) and a reference *E. coli* strain were tested. Sample sizes (AB/CB): ex vivo (11/14), non-stimulated (11/14), FR4930 (6/7), FR5730 (5/5), FR5333 (3/4), Tohama (1/3), *E. coli* (3/4). For statistical analyses, all *B. pertussis*-stimulated samples were pooled into a single group (AB, n=18 ; CB, n=23), regardless of isolate**.”

Comment 9: The labels for Fig 6B, 6C and 6D are incorrectly assigned on the figure as compared with the description in the legend, this should be corrected.

Response: We corrected labels for Fig 6B, C and 6D.

Comment 10: The loss of CD16 is described as a marker of granulocyte activation here, however loss of surface CD16 expression is associated with degranulation or apoptosis of neutrophils, with increased CD16 associated with activation (e.g. see PMID:23228566 and PMID:10380913). In addition, with the currently provided information, it is not clear whether this **shift in expression is due to a cell population shift or phenotype shift; CD66b+ CD16-** cells may reflect altered neutrophil states or an increased proportion of eosinophils which are CD16-. **Adding clearer labels to the flow gating strategy** displayed in Fig S3B and a complete list of marker composition for each cell in S3A (e.g. Treg: CD45+ CD3+ CD19- CD25+ CD127+) will provide necessary details for the reader to understand the possible cell populations included in each group. While additional discussion on the different mechanisms of reduced CD16 surface expression would also improve the manuscript.

Response: We thank the Reviewer for highlighting the important distinction between CD16 down-regulation associated with neutrophil degranulation/apoptosis versus CD16 up-regulation as a marker of activation. We fully agree that the initial wording in the manuscript could imply a simplified interpretation. To clarify that the reduced CD16 surface expression observed after *B. pertussis* stimulation is consistent with neutrophil degranulation or early apoptosis, rather than classical activation pathways, we have now revised the Results : **“Following 4-hour stimulation, granulocytes displayed a phenotype characterized by increased CD66b expression associated with a loss of CD16 surface proteins in AB and CB that could be consistent with neutrophil degranulation or early apoptosis (Fig 3C).”** and Discussion section **“Also, the reduced CD16 surface expression observed after *B. pertussis* stimulation suggest neutrophil degranulation or early apoptosis, rather than classical activation pathways.”**

We have updated S3B Fig with clearer and explicit gating labels for each population and have expanded S3A Fig to include the marker combinations used to define each immune subset.

Comment 11: The **methods used for determining pathway enrichment** should be provided.

Response: We agree with the reviewer. Pathway enrichment analysis was performed using a one-tailed Fisher’s exact test based on the hypergeometric distribution. We added it in the Methods section: **“Pathway enrichment analysis was performed using a one-tailed Fisher’s exact test based on the hypergeometric distribution.”**

Comment 12: The heatmap headers for Fig 5A and 6A are not consistent or easily understood, I suggest revising these.

Response: We changed the titles as follows: “**Fig 5. Changes in gene expression in CB after a 4-hour stimulation with *B. pertussis”*; “Fig 6. Genes differentially regulated between CB and AB after a 4-hour stimulation with *B. pertussis*”** and slightly modified the legend of Fig 5A and Fig 6A for clarification.
