# Supplementary material for: Modeling neonatal immune response to B. pertussis identifies early B cell activation and differentiation
Source: PLoS Pathog. 2026 Apr 22;22(4):e1014163. doi: 10.1371/journal.ppat.1014163 (PMC13167031; doi:10.1371/journal.ppat.1014163)
Supplement: Attachment — Submitted filename: Response to Reviewers_11032026-PL.docx [file ppat.1014163.s016.docx]

**PPATHOGENS-D-25-01905**

Thank you for your email and for your additional editorial comments on our manuscript. We have carefully addressed each of your points as detailed below.

**Additional Editor Comments:** Thank you for providing a substantially revised manuscript in response to concerns that were raised by the reviewers. You have effectively addressed the major points raised. As we discussed, there was a concern about one of your citations and you indicated that this would need to be corrected through a minor revision. Please submit a suitably modified version with that section re-written accordingly. Specifically, you were going to modify as shown below. “At baseline, ICAM levels were elevated, as previously described (Duffy et al., Immunity. 2014, PMID: 24656047). When cytokine concentrations from stimulated CB and AB were directly compared, 10 showed differential secretion (Fig 2B); of these, five were significantly upregulated upon stimulation: VEGF-A was found more elevated in AB whereas levels of IL-1a, IL-1b, IL-12p40 and TNF-a were higher in CB after stimulation with B. pertussis. IFNγ levels were higher in CB than in AB, but the stimulation-specific effect did not reach statistical significance, likely due to elevated baseline IFNγ levels and inter-donor variability (Biancotto et al, Plos one, PMID: 24348989; Duffy et al., Immunity. 2014, PMID: 24656047)."

**Response:** We have implemented the modifications described above.

**Journal Requirements:** If the reviewer comments include a recommendation to cite specific previously published works, please review and evaluate these publications to determine whether they are relevant and should be cited. There is no requirement to cite these works unless the editor has indicated otherwise.

**1)** In the online submission form, you indicated that Other raw data from our study are available upon reasonable request. All PLOS journals now require all data underlying the findings described in their manuscript to be freely available to other researchers, either
1. In a public repository
2. Within the manuscript itself
3. Uploaded as supplementary information.

This policy applies to all data except where public deposition would breach compliance with the protocol approved by your research ethics board. If your data cannot be made publicly available for ethical or legal reasons (e.g., public availability would compromise patient privacy), please explain your reasons by return email and your exemption request will be escalated to the editor for approval. Your exemption request will be handled independently and will not hold up the peer review process, but will need to be resolved should your manuscript be accepted for publication. One of the Editorial team will then be in touch if there are any issues.

**Response:** We added cytokines raw data as S2 dataset.

**2)**  Please ensure that the funders and grant numbers match between the Financial Disclosure field and the Funding Information tab in your submission form. Note that the funders must be provided in the same order in both places as well.

**Response:** We followed these recommendations.

**3)** Please ensure that the figure legend includes the proper citation indicating the figure was created using BioRender.

**Response:** We updated the figure legend (Fig 1) as requested.

**Figure resubmission:**

While revising your submission, we strongly recommend that you use PLOS’s NAAS tool ([https://ngplosjournals.pagemajik.ai/artanalysis](https://urldefense.com/v3/__https:/ngplosjournals.pagemajik.ai/artanalysis__;!!JFdNOqOXpB6UZW0!rmdud6dHX6uAWIw-O0G6wVPfL-TTGfOMnC4QFS3w3UWq156gLlycRSEJAQZ8NiVGNgbEI3FQd1TivgZML7mW$)) to test your figure files. NAAS can convert your figure files to the TIFF file type and meet basic requirements (such as print size, resolution), or provide you with a report on issues that do not meet our requirements and that NAAS cannot fix.

After uploading your figures to PLOS’s NAAS tool - [https://ngplosjournals.pagemajik.ai/artanalysis](https://urldefense.com/v3/__https:/ngplosjournals.pagemajik.ai/artanalysis__;!!JFdNOqOXpB6UZW0!rmdud6dHX6uAWIw-O0G6wVPfL-TTGfOMnC4QFS3w3UWq156gLlycRSEJAQZ8NiVGNgbEI3FQd1TivgZML7mW$), NAAS will process the files provided and display the results in the "Uploaded Files" section of the page as the processing is complete. If the uploaded figures meet our requirements (or NAAS is able to fix the files to meet our requirements), the figure will be marked as "fixed" above. If NAAS is unable to fix the files, a red "failed" label will appear above. When NAAS has confirmed that the figure files meet our requirements, please download the file via the download option, and include these NAAS processed figure files when submitting your revised manuscript.

**Response:** We used the NAAS tool provided by PLOS to convert our figure files and subsequently included the processed figures in the resubmitted manuscript.

**Reproducibility:**

To enhance the reproducibility of your results, we recommend that authors of applicable studies deposit laboratory protocols in protocols.io, where a protocol can be assigned its own identifier (DOI) such that it can be cited independently in the future. Additionally, PLOS ONE offers an option to publish peer-reviewed clinical study protocols. Read more information on sharing protocols at [https://plos.org/protocols?utm_medium=editorial-email&utm_source=authorletters&utm_campaign=protocols](https://urldefense.com/v3/__https:/plos.org/protocols?utm_medium=editorial-email&utm_source=authorletters&utm_campaign=protocols__;!!JFdNOqOXpB6UZW0!rmdud6dHX6uAWIw-O0G6wVPfL-TTGfOMnC4QFS3w3UWq156gLlycRSEJAQZ8NiVGNgbEI3FQd1Tivkg495Ek$)

**Response:** Thank you for this information. We will consider your suggestion to deposit laboratory protocol.
